# Biogenic selenium nanoparticles induce ROS-mediated necroptosis in PC-3 cancer cells through *TNF* activation

**DOI:** 10.1186/s12951-017-0276-3

**Published:** 2017-06-07

**Authors:** Praveen Sonkusre, Swaranjit Singh Cameotra

**Affiliations:** Institute of Microbial Technology, Sector 39 A, Chandigarh, 160036 India

**Keywords:** *Bacillus licheniformis* JS2, Biogenic selenium nanoparticles, PC-3, *TNF*, *IRF1*, Necroptosis, ROS, RIP1

## Abstract

**Background:**

Selenium is well documented to inhibit cancer at higher doses; however, the mechanism behind this inhibition varies widely depending on the cell type and selenium species. Previously, we have demonstrated that *Bacillus licheniformis* JS2 derived biogenic selenium nanoparticles (SeNPs) induce non-apoptotic cell death in prostate adenocarcinoma cell line, PC-3, at a minimal concentration of 2 µg Se/ml, without causing toxicity to the primary cells. However, the mechanism behind its anticancer activity was elusive.

**Results:**

Our results have shown that these SeNPs at a concentration of 2 µg Se/ml were able to induce reactive oxygen species (ROS) mediated necroptosis in PC-3 cells by gaining cellular internalization. Real-time qPCR analysis showed increased expression of necroptosis associated tumor necrotic factor (*TNF*) and interferon regulatory factor 1 (*IRF1*). An increased expression of RIP1 protein was also observed at the translational level upon SeNP treatment. Moreover, the cell viability was significantly increased in the presence of necroptosis inhibitor, Necrostatin-1.

**Conclusion:**

Data suggest that our biogenic SeNPs induce cell death in PC-3 cells by the ROS-mediated activation of necroptosis, independent to RIP3 and MLKL, regulated by a RIP1 kinase.

## Background

The treatment of cancer using nanoparticles is emerging as an alternative for cancer therapy. Nanoparticles provide site specific delivery of high drug load and thus reduce the risk of side effects and multidrug resistance in cancerous cells [1–3]. Since the drug delivery through NPs requires lower dose, it shows lower toxicity and offers increased half life to the carried drug molecule [4]. A diverse range of NPs have been synthesized and reported to have target specific enhanced anticancer activity. For example, the delivery of encapsulated PI3K inhibitor (BYL719) through fucoidan-based nanoparticles induced death in squamous cell carcinoma by preventing the side effects of hyperglycaemia [1]. Similarly, dendrimers or dendrimers- RNA triple helices conjugate were used as a nanoparticle to interact and adhere to the tumors for the specific delivery of miRNA [5, 6]. Likewise, gold, lipid and lipopeptide nanoparticles were used for siRNA or drug delivery [7–9]. Thiolated-PEG-COOH functionalized gold nanoparticles were also reported to deliver biohybrid RNAi-peptide specifically to the cancer cells [10]. Similarly, various functionalized gold, platinum nanoparticles, quantum dots, lipidated particles, liposomes, and dendrimers have been used extensively for targeted drug delivery, imaging and cancer cell killing [11–16]. Among these nanomaterials, selenium nanoparticles (SeNPs) are reported to be the most promising nanosystem which itself has high anticancer activity and better biocompatibility [17, 18]. Every form of selenium is reported to have some anticancer activity with a different mechanism of action, and most of them are reported for prostate cancer inhibition. Selenite is reported to trigger caspase-mediated apoptosis in association with DNA fragmentation, phosphorylation of JNK1/2 and p38 MAPK/SAPK2 along with mitochondrial superoxide generation in PC-3 cells [19, 20]. It is also reported to cause the G2/M cell cycle arrest and induction of apoptosis in HCT116 and SW620 colorectal carcinoma cells through Bax-dependent mitochondrial pathway [21]. Similarly, methyl seleninic acid stimulates apoptosis in DU-145 human prostate carcinoma cells via PARP cleavage [22]. Selenomethionine causes downregulation of Bcl-xL along with up-regulation of Bax, Bad, Bim, and caspase-9 activation in SW480 tumor model [23]. It is also reported to stimulate apoptosis through p53 dependent cell cycle arrest in HCT116 and RKO colon cancer cells [24]. Nevertheless, chemically synthesized nanosized selenium is shown to induce cell cycle arrest, at S phase, in HeLa cells [25]. It inhibits the growth of LNCaP cells by suppressing the expression of androgen receptors at both transcriptional and translational levels, causes phosphorylation, ubiquitination-mediated degradation of androgen receptors through Akt/Mdm2 mediated pathway [26]. Transferrin-conjugated SeNPs prompt intracellular ROS production and activate MAPKs pathways to induce p53-mediated apoptosis in MCF-7 cells [17]. Glucose decorated SeNPs also reported to induce apoptosis in HepG2, MCF-7, A549 and Neuro-2a cells [18].

Due to the similar biological efficacy of SeNPs with that of inorganic and organic selenium; anticancer therapy using such particles is currently an extensively studied area. To overcome the issue of the lower therapeutic index of these selenium compounds, and to achieve enhanced biocompatibility and greater stability compared to the chemically synthesized SeNPs with an eco-friendly approach [17, 26, 27], we synthesized SeNPs biologically from *Bacillus licheniformis* JS2 strain and studied their cytotoxic effects on human prostate epithelial adenocarcinoma cells, PC-3.

**Fig. 1:**
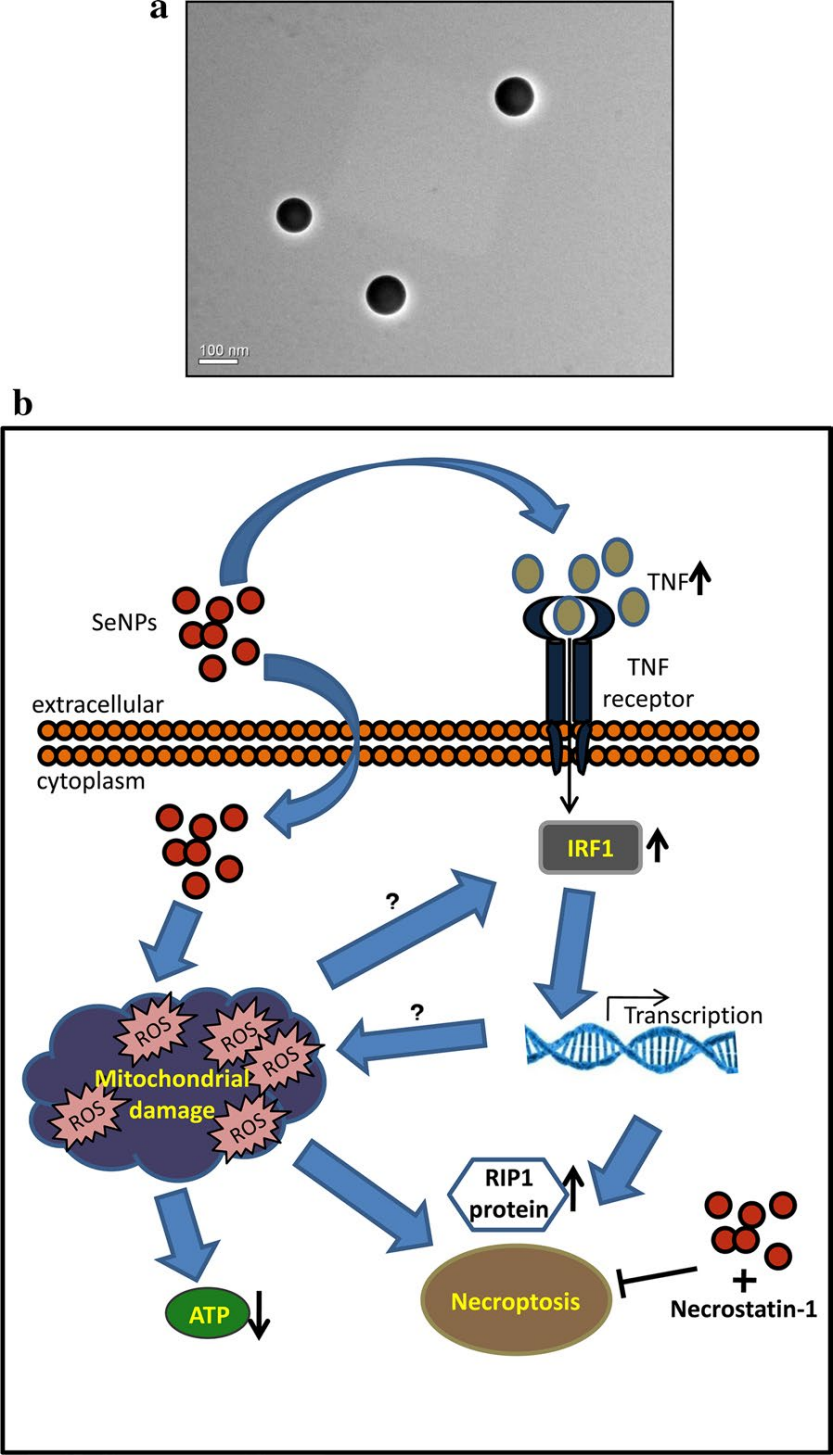
**a** TEM image of selenium nanoparticles extracted and purified from *B. licheniformis* JS2. Image was captured on a JEOL JEM 2100 TEM microscope at 200 kV. **b** Schematic representation of the proposed mechanism of selenium induced necroptosis in PC-3 cells. Exposure of SeNP to the PC-3 cells cause their cellular internalization and production of mitochondrial ROS which leads to ATP depletion and thus cell death. Exposure to SeNP also activates *TNF* and transcription factor, *IRF1*, responsible for the necroptosis through RIP1 protein. A treatment of necrostatin-1 along with SeNP prevents SeNP induced cell death

Earlier, we have demonstrated that our biogenic SeNPs of an approximate size of 110 nm in diameter (Fig. 1a), induce non-apoptotic cell death in these cancer cells without affecting the viability of primary cells [human peripheral blood mononuclear cells (hPBMCs)] [28]. However, the intrinsic details were still elusive. Here, we have investigated the detailed mechanism underlying the cell death pathway. We observed an excellent *TNF* and ROS-mediated necroptosis in these cells at a minimal concentration of 2 µg Se/ml of SeNP (Fig. 1b).

## Methods

### Microorganism and culture conditions

Selenium nanoparticle synthesizing bacteria, *Bacillus licheniformis* JS2, isolated from the selenium-contaminated agricultural soil of Nawanshahr district (latitude 31°07′ N and longitude 76°08′ E) of Punjab, India, was used to synthesize SeNP aerobically [29].

### Reagents

Tryptic soya broth (TSB) and agar (TSA) were procured from Hi-Media Laboratories. Sodium dodecyl sulfate (SDS), lysozyme, necrostatin-1, 3-(4,5-dimethylthiazol-2-yl)-2,5-diphenyltetrazolium bromide (MTT), metformin hydrochloride, 2-Deoxy-d-glucose, dihydrorhodamine 123, cytochalasin D, Durcupan™ ACM resin components; A, B, C, and D, triton X-100, absolute ethanol, and Bradford’s reagent were purchased from Sigma-Aldrich. 1-octanol, tris-buffer, chloroform, HCl, and luminata forte western HRP substrate were obtained from Merck-Millipore. Fetal bovine serum (FBS), TRIzol Reagent, and penicillin–streptomycin solution, and were purchased from Gibco-Invitrogen. CellTiter-Glo luminescent cell viability assay kit for ATP detection and CytoTox-ONE™ homogeneous membrane integrity assay kit for estimating LDH release were obtained from Promega and the manufacturer’s instructions were followed. DyNAmo ColorFlash SYBR Green qPCR kit and Verso cDNA synthesis kit were procured from Thermo Fisher Scientific. RIP1, RIP3, and β-actin antibodies were purchased from Cell Signaling Technologies, MLKL, pMLKL, and pRIP3 were procured from Abcam. Secondary HRP antibodies were obtained from Santa Cruz Biotechnology. All plastic wares for cell culture were obtained from Nunc. Millipore water (Type II) was used in all the experiments.

### Biosynthesis of selenium nanoparticle

*Bacillus licheniformis* JS2 strain was utilized for the synthesis of spherical SeNPs of an approximate size of 110 nm under aerobic condition in 1.8 mM sodium selenite stress. SeNPs were extracted and purified by following our previously reported method [28].

### Quantification of selenium

Overnight acid digestion of SeNPs in 3:1 nitric acid: perchloric acid solution was performed and the samples were analyzed in a Shimadzu AA-6800 atomic absorption spectrophotometer (AAS) with selenium cathode lamp. Samples were atomized on acetylene flame and the selenium was quantified at 196 nm wavelength.

### Cell lines and cell culture

A human prostate adenocarcinoma cell line (PC-3); derived from metastatic site, was purchased from the National Centre for Cell Science, Pune, India. Cells were cultured at 37 °C in a humidified incubator with 5% CO_2_ in Ham’s F-12K (Kaighn’s) medium supplemented with 10% fetal bovine serum, 50 units/ml streptomycin, and 100 units/ml penicillin.

### ATP depletion assay

PC-3 cells were seeded in 96-well opaque walled plate (white) at a density of 1 × 10^3^ cells per well in Ham’s F-12K (Kaighn’s) medium supplemented with antibiotics and 10% FBS and kept at rest for 24 h at 37 °C in a humidified 5% CO_2_ incubator. Cells were treated with SeNPs at a concentration of 2 µg Se/ml and incubated further for 6 and 12 h at 37 °C. Cells treated with 5 mM metformin and 1 mM 2-deoxy-d-glucose were used as positive control for necrosis. CellTiter-Glo™ reagent was used according to the manufacturer’s instructions to determine the levels of ATP present. The CellTiter-Glo™ Assay generates a “glow-type” luminescent signal, produced by the luciferase reaction. The amount of luminescent signal corresponding to the levels of ATP was determined on a GloMax^®^-96 Microplate Luminometer.

### Lactate dehydrogenase (LDH) release assay

PC-3 cells were seeded and kept on rest for 24 h as mentioned previously. Cells were treated with SeNPs at a concentration of 2 µg Se/ml and incubated for another 12, 18, 24, or 30 h at 37 °C. LDH release from the cells, an indicator of membrane damage, was quantified using CytoTox-ONE™ assay kit. The assay is based on the conversion of non-fluorescent resazurin into the fluorescent resorufin product, directly proportional to the amount of LDH present. The levels of LDH were determined in the form of fluorescent signals on a BioTek Power Wave Microplate reader.

### Dihydrorhodamine 123 (DHR123) staining and confocal microscopy

PC-3 cells were seeded onto sterile round 16 mm diameter glass coverslips in a 12-well tissue culture plate at a density of 2 × 10^5^ cells per coverslip in Ham’s F-12K (Kaighn’s) medium supplemented with antibiotics and 10% FBS. Cells were kept at rest for 24 h at 37 °C in a 5% CO_2_ incubator. After the rest period, cells were treated with SeNPs at a concentration of 2 µg Se/ml and incubated for 16 h. Both SeNP treated and untreated cells were stained with 1 µM DHR 123 for 30 min. After the incubation, the supernatant was discarded and the cells were washed 2–3 times with PBS (pH 7.4). Coverslips were placed inverted on the microscopic glass slides and visualized under Nikon A1R confocal microscope at 488 nm argon laser.

### DHR123 staining and flow cytometry

2 × 10^5^ cells per well were seeded in 12-well plates as detailed above. Cells were treated with SeNPs at a concentration of 2 µg Se/ml and incubated for different time intervals, viz., 6, 9, 12, 15, 18, 21, or 24 h. Cells were stained with 1 µM dihydrorhodamine 123 for 30 min, followed by harvesting with 10 mM EDTA solution. Harvested cells were washed twice with PBS (pH 7.4) and acquired in a BD AccuriC6 Flow Cytometer (BD Biosciences). The data analysis was performed by FlowJo software.

### Cytochalasin D treatment

PC-3 cells were seeded in 12-well plates at a density of 2 × 10^5^ cells per well as mentioned previously. After 24 h, test wells were treated with 2 µM cytochalasin D for 30 min. The medium was discarded; adhered cells were rinsed and supplemented with 1 ml of fresh Ham’s F-12K (Kaighn’s) medium. Cells were treated with SeNPs at a concentration of 2 µg Se/ml and incubated for 16 h at 37 °C in a CO_2_ incubator. Cells were harvested, and an AnnexinV-FITC Apoptosis Detection Kit was used to stain the cells with FITC labeled annexinV and propidium iodide according to the manufacturer’s instructions. Cells were acquired in a BD AccuriC6 Flow Cytometer (BD Biosciences). The data analysis was performed by FlowJo software.

In another set, cells were stained with 1 µM dihydrorhodamine 123 for 30 min before harvesting. Harvested cells were washed 2–3 times with PBS (pH 7.4) and acquired in a BD AccuriC6 Flow Cytometer (BD Biosciences). The data analysis was performed by FlowJo software.

### Block preparation and TEM analysis

To confirm the cellular internalization of SeNPs, TEM analysis of PC-3 cells was performed after 12 h treatment with SeNP. PC-3 cells were seeded in a 6 well plate at a density of 5 × 10^5^ cells per well and kept at rest for 24 h at 37 °C. After the rest period, cells were treated with SeNPs at a concentration of 2 µg Se/ml, and incubated for 12 h. Cells were harvested using 10 mM EDTA solution and washed thrice with PBS. 700 µl of Karnovsky’s fixative was added and the samples were fixed for 3 h at 4 °C. Samples were stained, dehydrated, and embedded as per the standard protocol using Durcupan™ ACM resin components; A, B, C and D (Sigma-Aldrich). Ultrathin sections of 70 nm thickness were prepared using a Leica EM UC7 ultramicrotome. Samples were collected on copper grids and visualized at 200 kV on a JEOL JEM-2100 TEM microscope after negative staining.

### RNA extraction

PC-3 cells were seeded in 6-well plates at a density of 5 × 10^5^ cells as mentioned earlier. After the rest period, cells were treated with SeNPs at a concentration of 2 or 4 µg Se/ml and incubated for 16 h. Cells were rinsed with PBS and harvested using TRIzol reagent. Harvested cells were lysed by multiple pipetting (15–20 times) followed by 5 min incubation at RT. 150 µl of chloroform was added, mixed by inversion (approx. 15 times) and kept undisturbed at RT for another 5 min. Samples were centrifuged at 10,000×*g* for 15 min at 4 °C. Approximate 200 µl of the upper aqueous layer from each microcentrifuge tube (MCT) was carefully transferred to the respective fresh diethyl pyrocarbonate (DEPC) treated MCT and an equal volume of isopropanol was added. Samples were gently mixed by inverting the tubes 4–5 times followed by 10 min incubation at room temperature (RT). After the incubation, RNA-containing samples were centrifuged at 14,000×*g* for 10 min at 4 °C. Supernatants were discarded and the pellets were washed twice with 70% ethanol at 10,000×*g* for 5 min at 4 °C. Each MCT containing the RNA pellet was allowed to dry at RT, and the dried pellets were resuspended in 20 µl of nuclease free water.

### cDNA synthesis and quantitative Real Time PCR

Extracted RNA were quantified on nanodrop (Thermo Scientific), 1000 ng of each sample was reverse transcribed using a Verso cDNA synthesis kit (Thermo Fisher Scientific) according to the manufacturer’s instruction. cDNA was amplified by Eppendorf MasterCycler RealPlex^4^ PCR with gene specific primers using the DyNAmo ColorFlash SYBR Green qPCR kit (Thermo Fisher Scientific). Housekeeping gene, β-actin, was used as an internal control. The relative fold change was calculated by using formula 2^−∆∆Ct.^

### Necroptosis inhibition

Necrostatin-1 was used to inhibit SeNPs induced necroptosis in PC-3 cells. 3.5 × 10^3^ cells per well were seeded in Ham’s F-12K (Kaighn’s) medium in 96-well flat bottom cell culture plates. After the resting period of 24 h, cells were subjected with 2 µg Se/ml SeNPs or, 2 µg Se/ml SeNPs and necrostatin-1 (20 μM, or 50 μM) or DMSO, and cultured for 24 h at 37 °C. 10 µl of (5 mg/ml) MTT [3-(4,5-dimethylthiazol-2-yl)-2,5-diphenyltetrazolium bromide)] solution was added to each well, and the plates were incubated for 3.5 h at 37 °C. 80 µl of the solubilizing solution, 20% SDS (w/v) in 50% DMF (v/v), was added to each well in a sterile condition. The plates were kept at 37 °C for 3 h at 120 rpm. 130 µl from each well was transferred into a fresh 96 well plate and analyzed on a BioTek Power Wave Microplate reader at 570 nm. Production of the violet colored formazan in this assay corresponds to the cell viability.

### Protein extraction and western blotting

PC-3 cells were seeded in 6-well plates at a density of 5 × 10^5^ cells as mentioned earlier. Cells were then subjected with SeNPs at a concentration of 2 µg Se/ml, or 4 µg Se/ml, or 2 µg Se/ml with Nec-1 (50 μM), or 4 µg Se/ml with Nec-1 (50 μM), and incubated for 12 h. Cells were rinsed with PBS and harvested in cell lysis buffer [150 mM NaCl, 50 mM Tris–Cl (pH 7.4), 2 mM EDTA, 1 mM dithiothreitol (DTT), 4 mM Na_3_VO_4_, 1% triton-X 100, and 1% glycerol], containing protease inhibitor cocktail. Protein content was estimated using Bradford’s reagent. 30 µg protein samples were separated on 10% sodium dodecyl sulfate–polyacrylamide gel electrophoresis (SDS-PAGE) followed by protein transfer to polyvinylidene difluoride (PVDF) membrane. After blocking with 5% BSA, membranes were incubated overnight with primary antibodies (anti-MLKL, anti-phospho MLKL, anti-phospho RIP3, anti-β-actin, anti-RIP1, and anti-RIP3 antibodies), followed by incubation with HRP-conjugated secondary antibodies. Luminata Forte Western HRP substrate (Millipore) was used for the blot development.

### Statistical analysis

All the experiments were performed at least in triplicates and presented here as their mean ±SD. GraphPad Prism 6 software was used for all the statistical analysis. Statistical significances were calculated using the unpaired Student’s *t* test.

## Results

### Biogenic SeNPs causes mitochondrial damage without affecting the cell membrane integrity

**Fig. 2:**
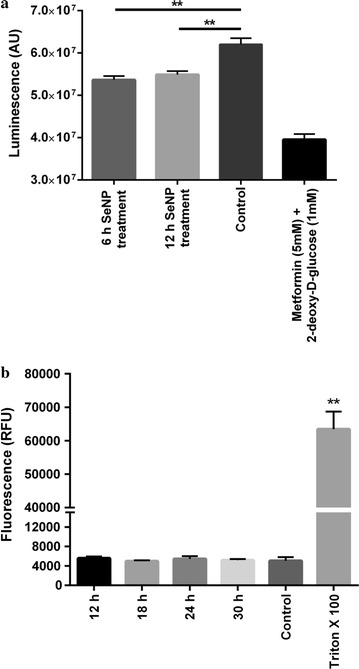
**a** PC3 cells treated with 2 µg Se/ml SeNPs for 6 or 12 h showed a significant decrease in the levels of cellular ATP compared to the control cells. Metformin and 2-deoxy-d-glucose treated cells were taken as a positive control. ATP was quantified using CellTiter-Glo™ reagent. The intensity of luminescence was proportional to the quantity of ATP present in the sample. The experiment was conducted in triplicate. **p < 0.01 represents a significant difference in the ATP level. **b** Cells treated with 2 µg Se/ml SeNPs for 6, 12, 18, 24, or 30 h showed no LDH release in the culture medium compared to the negative control (PBS treated cells). Triton-X 100 treated cells were taken as a positive control. The intensity of fluorescence was directly proportional to the quantity of LDH present in the sample. The experiment was conducted in triplicate. **p < 0.01 represents a significant difference in the LDH levels of positive control and SeNP treated cells

Intracellular ATP levels were determined after SeNP treatment. Cells treated for 6 or 12 h with 2 µg Se/ml SeNP show a significant decrease in the levels of cellular ATP compared to the control cells, suggesting the mitochondrial damage (Fig. 2a).

The levels of LDH were also estimated by using CytoTox-ONE homogeneous membrane integrity assay kit (Promega). No significant increase in the LDH level was observed in the culture medium after treating cells with 2 µg Se/ml SeNP for 12, 18, 24, or 30 h compared to the untreated cells (Fig. 2b).

### SeNP stimulates ROS production after gaining cellular internalization

**Fig. 3:**
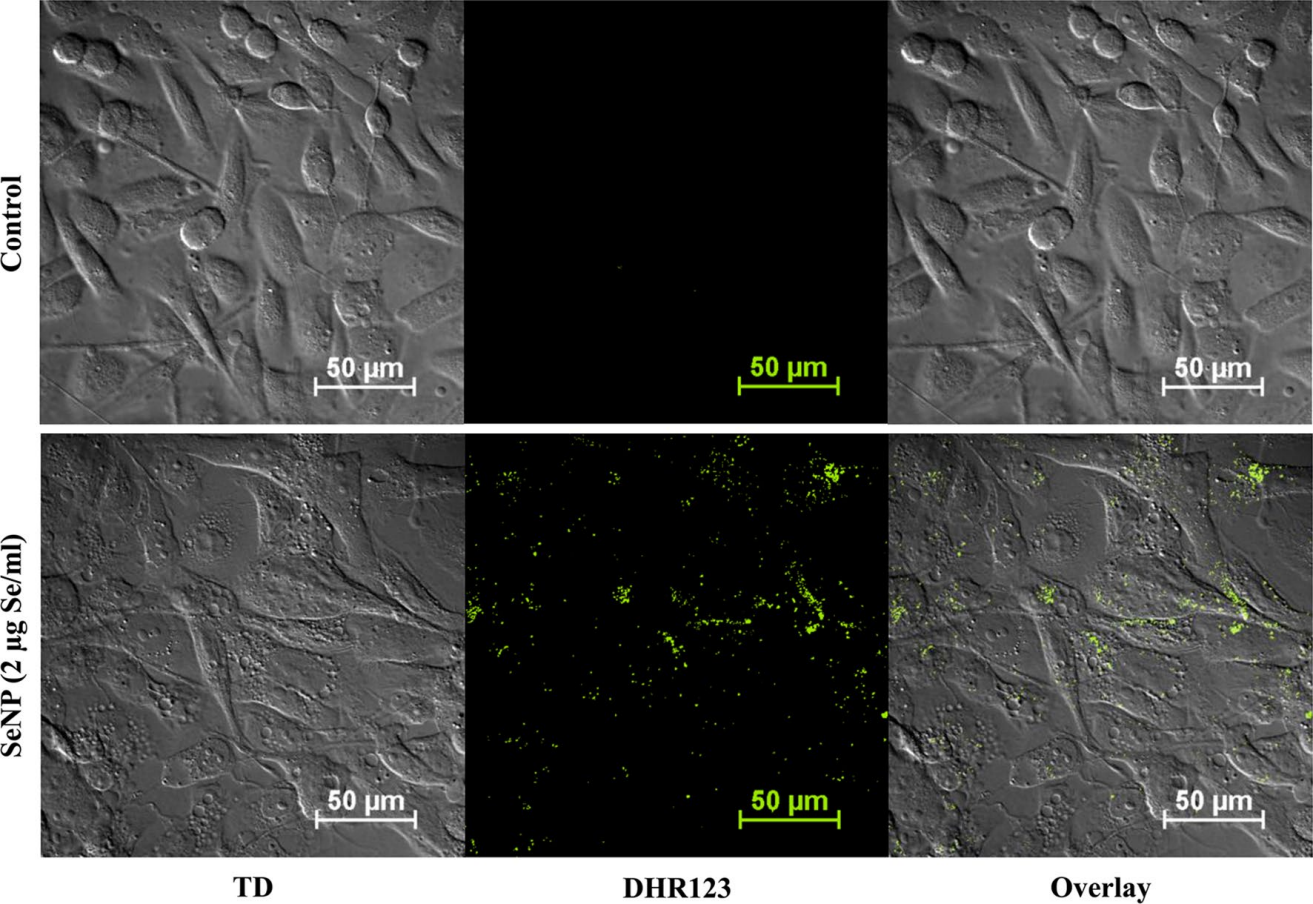
Biogenic SeNPs induce ROS-mediated cytotoxicity in PC-3 cancer cells. 24 h cultured PC-3 cancer cells were treated with SeNPs at a concentration of 2 µg Se/ml for 12 h. Mitochondrial ROS were visualized using dihydrorhodamine 123 at 60× oil immersion under the confocal microscope with 488 nm argon laser. ROS-induced cleavage of dihydrorhodamine 123 produces a fluorescent molecule, rhodamine 123, which was evident in the form of* greenish yellow *colored puncta

As selenium is reported to cause oxidative stress, levels of reactive oxygen species (ROS) were measured at different time intervals after SeNP treatment using a non-fluorescent molecule dihydrorhodamine 123. Dihydrorhodamine 123 which can passively diffuse across the membrane converted into a fluorescent probe rhodamine 123 in the presence of ROS and localize into the mitochondria which were detected by confocal microscopy and quantified in FACS. Confocal microscopy performed after 12 h of SeNPs treatment showed the presence of fluorescent rhodamine 123 in the form of puncta as an indicator of mitochondrial ROS (Fig. 3).

**Fig. 4:**
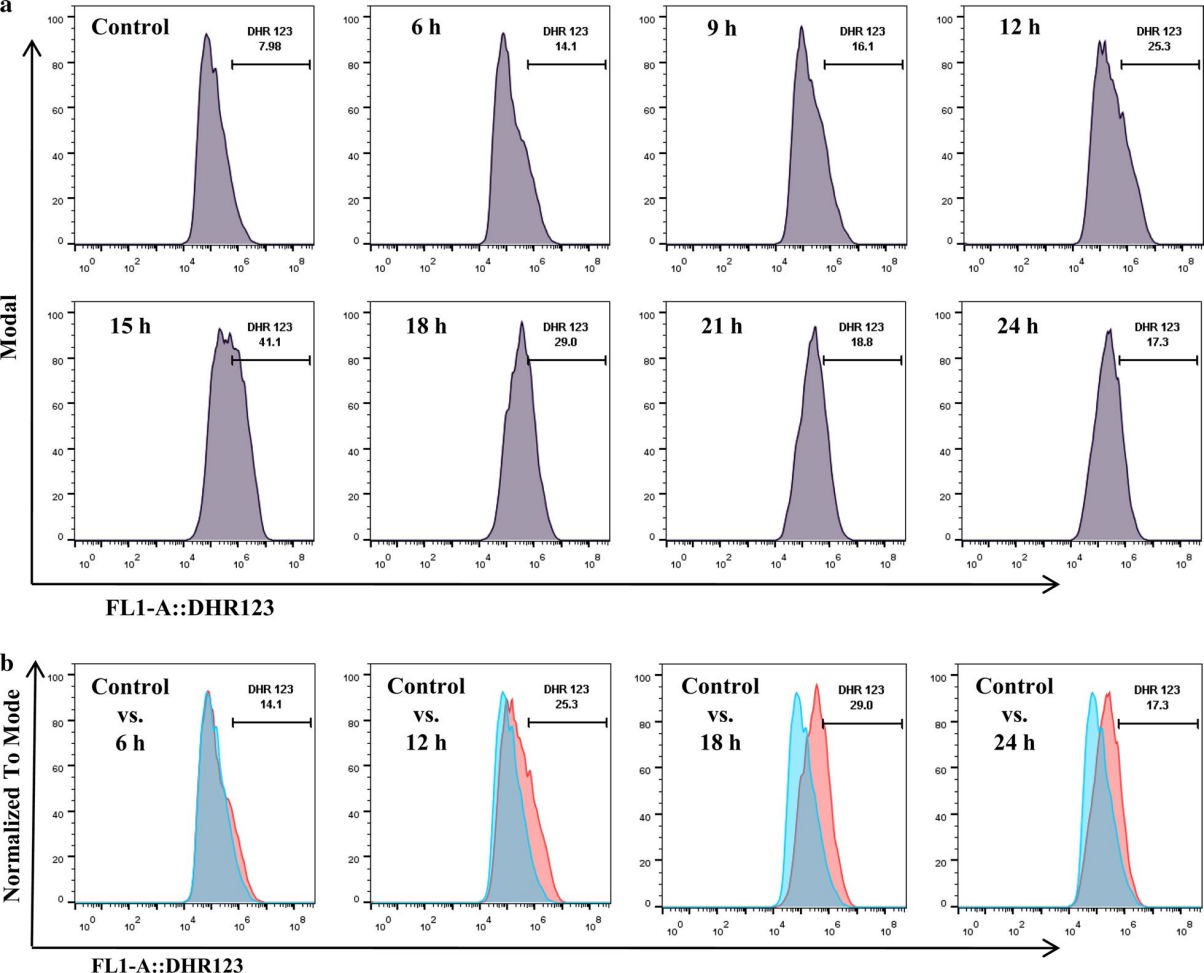
**a** FACS results showing the time-dependent increase in the ROS production after 2 µg Se/ml SeNP treatment for 6, 9, 12, 15, 18, 21 or 24 h. DHR 123 was used as a mitochondrial ROS indicator. A clear shift in the cell population with maximum ROS production was observed after 15 h of treatment. **b** Comparative analysis of the shift in the PC-3 cell population after 6, 12, 18 or 24 h of treatment with SeNPs. Experiments were performed in triplicate

The time-dependent change in the levels of mitochondrial ROS was also estimated via FACS. Results indicated that the production of ROS increased gradually, it was maximum at 15 h of treatment and then decreased gradually as the number of viable cells decreased (Fig. 4a). A clear shift in the cell population was started appearing after 12 h of treatment in comparison to untreated cells (Fig. 4b).

**Fig. 5:**
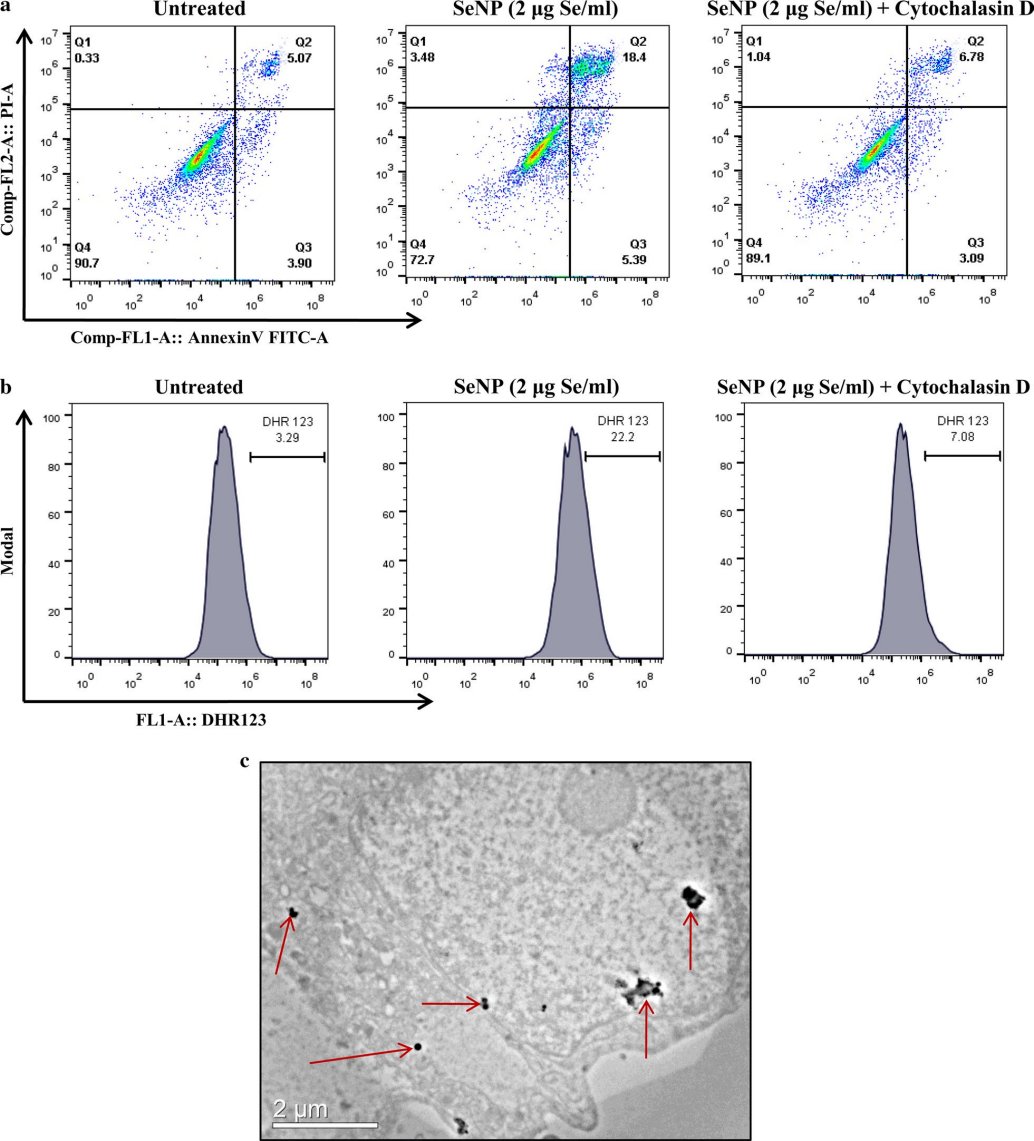
Endocytosis of SeNPs induces ROS-mediated cell death. **a** Cytochalasin D treated and untreated cells cultured in the presence of SeNPs (2 µg Se/ml) for 16 h showed more viability in cytochalasin D treated cells on AnnexinV-PI staining. **b** Staining with DHR123 also showed insignificant ROS production in cytochalasin D-treated cells. **c** TEM image of a PC-3 cell, acquired after 12 h treatment with SeNPs, showing the cytoplasmic localization of NPs. All the experiments were performed in triplicate

To confirm that the cytotoxicity is caused by the cellular internalization (endocytosis) of SeNPs, cells were treated with cytochalasin D (an inhibitor of actin polymerization which is known to block endocytosis and >90% of the phagocytosis [30]) prior to SeNP treatment. FACS analysis of AnnexinV-FITC and propidium iodide or DHR123 stained cells after 16 h of SeNP treatment showed more viability with less ROS production in cells treated with cytochalasin D, compared to cytochalasin D untreated cells (Fig. 5a, b). This suggested that toxicity caused by the selenium nanoparticles in PC-3 cells is due to their cellular internalization.

Endocytosis of SeNPs was confirmed by visualizing their intracellular localization under transmission electron microscope (TEM) by preparing ultrathin sections of SeNP treated PC-3 cells using an ultramicrotome. NPs were mostly found to be located in the cytoplasm (Fig. 5c).

### SeNP induces overexpression of necroptotic genes

**Fig. 6:**
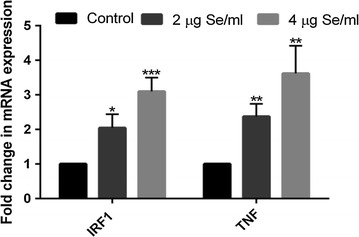
Real-time gene expression profiling of SeNP treated and untreated PC-3 cells. A significant dose-dependent fold change was observed in the expression of *IRF1* and *TNF* gene after SeNP treatment. The results were verified by three repetitions of experiments and each experiment was conducted in triplicate. *p < 0.05, **p < 0.01, and ***p < 0.001 represents a significant change in mRNA expression

To identify the pathway involved in cell death, real-time mRNA expression analysis was performed. Expression of characteristic molecules from different cell death pathway was studied in a real-time PCR. A dose-dependent overexpression of only *TNF* and *IRF1* mRNA was observed in SeNP treated cells. More than twofolds or fourfolds increase was observed in the expression of *TNF* and *IRF1* mRNA under 2 µg or 4 µg Se/ml SeNP stress, respectively (Fig. 6).

### SeNP induces RIP3 independent necroptosis in PC-3 cells

**Fig. 7:**
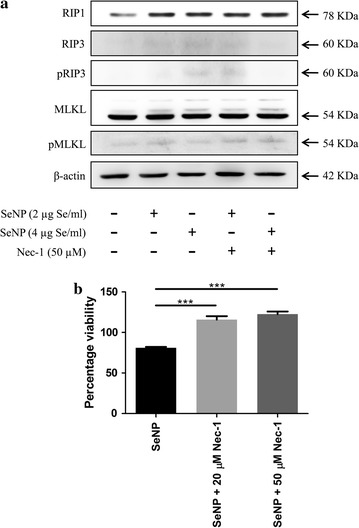
**a** Western blot analysis of necroptosis associated proteins. A significantly elevated level of RIP1 protein was observed after 12 h treatment with SeNP compared to the untreated cells signifies its overexpression or stabilization upon SeNP treatment. However, no change was observed in RIP3 and MLKL protein levels. Similarly, the presence of Nec-1 did not affect expression pattern of any of the protein. **b** PC-3 cells were cultured in the presence of 2 µg Se/ml SeNPs and/or Nec-1 for 24 h. Cell viability was determined by MTT assay. Significantly more viable cells were observed in wells treated with SeNP along with Nec-1. The results were verified by three repetitions of experiments and each experiment was conducted in triplicate. ***p < 0.001 represents a significant difference in the PC-3 cell viability treated with SeNP or SeNP with Nec-1

Since necroptosis is reported to be coupled with the phosphorylation of RIP3 and MLKL, we performed western blot analysis to estimate the phosphorylated and unphosphorylated status of these proteins. Results showed no expression of RIP3 as well as its phosphorylated form in both SeNP treated and untreated cells. Furthermore, no change in MLKL expression and the absence of its phosphorylated form was observed in control as well as SeNP treated cells. However, SeNP dependent significant increase in the RIP1 protein expression was evident. The treatment of Nec-1 along with SeNP did not affect expression pattern of any of the protein (Fig. 7a).

The viability of SeNP treated cells cultured in the presence or absence of necroptosis inhibitor, Necrostatin-1, was determined in an MTT assay. A significant relative increase in the cell viability was observed in wells supplemented with Nec-1. The viability was directly proportional to the concentration of Nec-1 added (Fig. 7b).

## Discussion

Every form of selenium has more or less anticancer activity and it is typically observed in prostate, colon and lung cancer [31–37]. However, the anticancer properties depend on the selenium species, dose, cancer type and stage [32]. It can be affected by the environmental factors, genotype and the bioavailability of selenium. The mode and extent of cell death vary widely depending on the selenium species. Several mechanisms have been suggested for the anticancer activity of selenium, including cell cycle arrest, antioxidation, apoptosis, interruption of cell signaling pathway [17–20, 25, 38] etc. Recently, SeNPs came into limelight because of their excellent anticancer activity with lower toxicity compared to the other inorganic and organic forms of selenium [27, 39–41] and thus, emerging as a potential cancer chemopreventive agent.

In our previous study, we have demonstrated that a minimum concentration of only 2 µg Se/ml of these, ~110 nm in diameter, well characterized, *B. licheniformis* derived, sterically stabilized SeNP is very effective in inhibiting the proliferation and inducing mortality in PC-3 cancer cells through a caspase-independent pathway [28].

In this present report, we have analyzed the underlying mechanism behind the SeNP mediated PC-3 cell death. A significant depletion in the ATP levels was observed within 6 h of SeNP treatment, supporting the phenomenon of necrosis/necroptosis [42]. As LDH release is a hallmark feature of necrotic/necroptotic cell death, we have studied the LDH release upon SeNP treatment. Surprisingly, there was no LDH release observed in the culture medium even after 30 h of treatment.

We also observed endocytosis of SeNPs that leads to the drastic production of mitochondrial ROS. ROS generation along with ATP depletion indicated the SeNP induced mitochondrial damage. A significant increase in the levels of *TNF* and *IRF1* gene expression was also observed in qPCR. *IRF1* is a well-known transcription activator of the genes induced by interferons α, β, and γ thereby inhibits the cell growth and suppresses tumor progression. Previous studies suggested that production of ROS and the activation of *TNF* and *IRF1* genes are involved in the induction of regulated necrosis (necroptosis) [43–46]. Reactive oxygen species of mitochondrial origin are also extensively documented in necroptotic cell death [46–48]. Conversely, glucose decorated and transferrin conjugated SeNPs were reported to cause apoptosis by the induction of caspase 3, 8 and 9 and mitochondrial ROS. However, in this study PC-3 cells did not show any sign of apoptosis under SeNPs treatment. This is probably because our SeNPs have different surface, size and structural properties compared to the earlier reports. SeNPs are reported to have different mechanisms of cell death depending on the mode of synthesis, the presence of functional groups, bioavailability, size, structure (lattice arrangement of atoms), and compactness of the NPs [17, 18, 25–27]. This variability is largely observed when chemical and biological syntheses of NPs are compared. These changes play a tremendous role in their biological activity [49–52].

According to the literature, the conventional pathway of necroptosis is triggered by a number of inflammatory signals like TNF α and TLRs [53]. The process is independent of caspases and is initiated through a necrosome complex containing RIP1 and RIP3 kinases. Necroptotic cell death is dependent on the phosphorylation of MLKL through RIPK3. MLKL is an essential necroptosis effector molecule downstream to the RIP1/RIP3 complex, which after phosphorylation migrates and localized to the plasma membrane and compromises its integrity that in turn releases intracellular proinflammatory molecules [54–56].

However, our western blot results showed no expression of RIP3 and no MLKL phosphorylation. This declined the possibility of necrosome formation and indicates that biogenic SeNP induced cell death is not activated through a conventional RIP3–MLKL necroptosis pathway. In support of our results, the literature also suggests, most of the cancer cell lines, including the PC-3 cells, do not express RIP3 due to the methylation-dependent gene silencing [57, 58]. However, we have observed a significant increase in RIP1 expression at the protein level after SeNP treatment. Though, a similar change was not observed at mRNA level (date not shown), suggesting that probably SeNP treatment is stabilizing the RIP1 protein, responsible for the cell death, possibly due to post-translational modification(s) which can be explored further.

We also observed no effect of Nec-1 on the MLKL, pMLKL, RIP1, RIP3, and pRIP3 expression. However, a significant increase in the cell viability was observed on Nec-1 treatment, suggesting the role of RIP1 in SeNP induced cell death. In support of our results, RIP3 and MLKL independent necroptosis is also reported in the literature [59–61].

Here, necroptosis does not involve pMLKL, and probably because of which we have not observed membrane damage. As per our knowledge, necroptosis event with the formation of RIP1/RIP3 complex is shown only in the RIP3 expressing cells. Here we suggest the possibility of SeNP induced RIP3/MLKL independent necroptosis downstream to the RIP1 in RIP3 non-expressing PC-3 cells.

## Conclusion

We summarize our study with a conclusion that *B. licheniformis* derived sterically stabilized biogenic SeNP at a minimum concentration of 2 µg Se/ml cause *TNF* and *IRF1* induced ROS-mediated necroptosis, in PC-3 cells. The event is observed dependent to the RIP1, however, independent of the RIP3 and the activation of MLKL and thus independent of the necrosome complex formation. Further studies can be done to identify the events and molecules involved in this necroptosis pathway mediated by the RIP1 kinase.

## Abbreviations

AAS: atomic absorption spectrophotometer
ATP: adenosine triphosphate
BSA: bovine serum albumin
DEPC: diethyl pyrocarbonate
DHR 123: dihydro rhodamine 123
DMF: dimethylformamide
EDTA: ethylenediaminetetraacetic acid
FACS: fluorescence activated cell sorting
FITC: fluorescein isothiocyanate
HRP: horseradish peroxidase
IRF1: interferon regulatory factor 1
LDH: lactate dehydrogenase
MCT: microcentrifuge tube
MLKL: mixed lineage kinase domain-like protein
MTT: 3-(4,5-dimethylthiazol-2-yl)-2,5-diphenyltetrazolium bromide
Nec-1: necrostatin-1
PBMCs: human peripheral blood mononuclear cells
pMLKL: phosphorylated form of MLKL
pRIP3: phosphorylated form of RIP3
PVDF: polyvinylidene difluoride
qPCR: quantitative real-time PCR
RIP1: receptor-interacting serine/threonine-protein kinase 1
RIP3: receptor-interacting serine/threonine-protein kinase 3
ROS: reactive oxygen species
RT: room temperature
SD: standard deviation
SDS-PAGE: sodium dodecyl sulfate–polyacrylamide gel electrophoresis
SeNP: elemental selenium nanoparticles
TSA: tryptic soya agar
TSB: tryptic soya broth
TEM: transmission electron microscope
TNFα: tumor necrotic factor alpha
TLRs: toll-like receptors
